# Injury Characteristics Among Japanese International Athletes: Report on the Pre-competition Medical Check-Up Data of the Japanese Olympic Committee

**DOI:** 10.7759/cureus.72869

**Published:** 2024-11-02

**Authors:** Naoko Fukuda, Mika Hangai, Ritsuko Hashimoto, Yusuke Nishida, Yuri Mizutani, Toru Okuwaki, Kohei Nakajima

**Affiliations:** 1 Department of Sports Medicine, Japan Institute of Sports Sciences, Tokyo, JPN; 2 Department of Orthopaedic Sugery, Sensory and Motor System Medicine, Graduate School of Medicine, The University of Tokyo, Tokyo, JPN

**Keywords:** asian, athlete, elite athlete, epidemiology, injury, japanese, olympics, sex-stratified comparison, youth athlete

## Abstract

Introduction

Injury trends among international athletes across sports remain underexplored in out-of-competition settings, particularly among Asians. The aim of this descriptive epidemiological study is to investigate the characteristics of injuries among Japanese international athletes during pre-competition medical check-ups from 2008 to 2019.

Methods

We analyzed the medical check-up data of candidates for international multi-sport events according to the International Olympic Committee consensus statement. At the medical check-up, athletes' injuries were categorized into two groups based on clinical assessment. "Injuries" refer to conditions that necessitate immediate treatment or further detailed examination. On the other hand, "complaints" encompass both such "injuries" and conditions for which treatment has already commenced, allowing athletes to continue participating in competitions or training while still requiring ongoing medical monitoring. The cohort was categorized into youth and adult groups, with adults defined as those aged ≥18 years.

Results

Overall, 10,854 athletes (4,966 females, 45.8%; 5,888 males, 54.2%; median age 22.0 {20.0-25.0} years, 56 sports) were enrolled; 2,333 “injuries” were registered (21.5 “injuries” per 100 athletes). The “injury” prevalence was 16.2% (95% CI, 0.16-0.17) and significantly associated with females (odds ratio {OR} 1.21; 95% CI, 1.09-1.34) and adult group (OR, 1.35; 95% CI, 1.08-1.69) based on binomial logistic regression analysis. Of a total of 10,027 “complaints” (92.4 “complaints” per 100 athletes), the “complaint” prevalence was 55.3% (95% CI, 0.54-0.56) and higher in females (OR, 1.44; 95% CI, 1.33-1.55) and adult group (OR, 1.50; 95% CI, 1.29-1.75). Stratified by sport, male soccer players had a higher “injury” prevalence than females (95% CI, 0.45-0.98), whereas females had a higher “injury” prevalence in hockey (1.70-7.29) and fencing (1.12-5.44). The “complaint” prevalence was higher in females for athletics, skiing, swimming, hockey, judo, badminton, fencing, water polo, weightlifting, and golf. There was no significant difference between the sexes in other sports. The knee (“injury,” 20.1%; “complaint,” 20.2%), lumbosacral (15.5%; 17.0%), ankle (13.0%; 15.4%), and shoulder (13.0%; 12.1%) were most commonly affected. The injury proportion ratio for the ankle was “injury”/“complaint” 0.82 (95% CI, 0.72-0.94), with the ankle “complaint” proportion being higher than “injury.” When stratified by injury location and sex, knee “injury” was more common in males (206 in females vs. 262 in males; 95% CI, 0.59-0.88), whereas ankle “complaint” was more common in females (842 in females vs. 700 in males; 95% CI, 1.04-1.29).

Conclusion

This is the first cross-sectional report of injuries in Asian international athletes outside of competition periods. Injury prevalence was higher in females than in males and in adults than in youths. Sex differences in injury varied by site and severity. These findings may suggest the need for more tailored injury prevention and performance support strategies for international competitions.

## Introduction

Systematic injury surveillance forms the basis for developing preventive measures in sports. Previous studies have predominantly focused on injuries occurring during competitions (single-sport tournaments or multi-sport events) or have investigated incidence within a single sport over time [1-3]. Various across-sport surveillance studies of elite athletes have reported on injury incidence during competitions by the International Olympic Committee (IOC) [4-7]. An expert group convened by the IOC has established an injury surveillance system for multi-sport events [8] that has been used at major international competitions such as the Olympic Games and World Championships. In contrast, the Canadian Intercollegiate Sports Injury Surveillance System [9] and the National Collegiate Athletic Association Injury Surveillance System [10,11] monitor within-season injuries across sports. Although injury-reporting systems have been established for specific injury types, little is known of injury trends during the normal training and preparatory phases, except for short periods in major Olympic-level international competitions, with some exceptions such as football.

Our sports clinic at the Japan Institute of Sports Sciences (JISS) has been providing medical examinations, including internal medicine, orthopedics, and dentistry, to national athletes across various sports in Japan since its establishment in 2001. Participants at international multi-sport events such as the Summer and Winter Olympic Games, Asian Games, East Asian Games, Universiade, and Youth Olympic Games undergo mandatory medical check-ups stipulated by the Japanese Olympic Committee (JOC) within six months prior to the tournament.

Although several international sports federations mandate medical examinations, the specific criteria and assessment items vary by sport. Accordingly, no study has uniformly assessed the medical check-up data for elite athletes collected by the National Olympic Committees across competitions.

In order to better tailor injury prevention measures, this study aimed to describe the injury characteristics of elite athletes in Japan.

## Materials and methods

Study design

In this cross-sectional study, we retrospectively evaluated medical data in accordance with the IOC Consensus Statement [5,12] for the preparatory phases outside the short period of major international multi-sport events.

Clinical assessment

The medical check-up was conducted by skilled orthopedic specialists certified as domestic sports specialists. When necessary, imaging tests such as ultrasound (US), plain X-rays, CT, and MRI were employed to ensure detailed and accurate diagnosis. The orthopedic surgeons recorded the site classification, left and right sides, and diagnoses. Each diagnosis was categorized into one of three levels: “active,” requiring further treatment and scrutiny; “follow-up,” where treatment had been initiated and follow-up was required; and “inactive,” where the patient was already cured.

The study was reviewed by the Ethical Committee of the JISS (2023-032, approved January 29, 2024). Prior to the study, all athletes provided informed consent for the scientific use of their medical check-up data.

Data collection

Candidate athletes from the Summer and Winter Olympic Games, Asian Games, East Asian Games, Universiade, and Youth Olympic Games held between 2008 and 2019, with a total of 30 games and 56 competitions, were included. We compiled a database of all registered injury diagnoses from medical check-up records aggregated from multiple cross-sectional surveys. Previous diagnoses were classified according to the IOC consensus statement [12], as described by Bahr et al. (head, face, neck, shoulder, upper arm, elbow, forearm, wrist, hand, chest, thoracic spine, lumbosacral, abdomen, hip/groin, thigh, knee, lower leg, ankle, foot, and region unspecified). The diagnoses were reorganized into uniform notations and reviewed. Diagnoses that were recorded bilaterally were modified and separately registered for the left and right sides, as appropriate. For example, bilateral patellar tendinopathy was registered as right and left patellar tendinopathy. From the database, we extracted information on active and follow-up diagnoses.

Operational definitions

Given that “active” and “follow-up” diagnoses are considered problematic, we defined “active” diagnosis as “injury,” while the sum of both “active” and “follow-up” injuries as "complaint.” We consider the clinical state of injury to be an important evaluation indicator when determining an athlete's participation in competition and estimating the impact on their performance. For this reason, we analyzed the clinical problems of athletes into two categories; we defined “active” classified in the aforementioned clinical assessment as “injuries” and added “follow-up” to “active” as “complaints.” We examined the prevalence of “injuries” and “complaints” by sex and age group. Generational stratification included a youth group (age <18 years) and an adult group (age ≥18 years). Sex differences were examined by sport and site, as well as in the overall study population. Comparisons by injury site were undertaken for the four sites with the highest number of registrations and were based on the “injury” to “complaint” ratio.

Statistical analysis

The study population consisted of athletes who underwent medical check-ups. If an athlete attended more than two medical examinations, each visit was counted as a separate athlete. The prevalence of “injuries” and “complaints” was analyzed per athlete, with each athlete recorded as having one injury, irrespective of the number of injuries sustained.

Pearson's chi-squared test and Fisher’s exact test were used to determine differences in binary variables, and unpaired Student’s t-tests were used to compare continuous variables. P < 0.05 indicated significant intergroup differences. Overall prevalence was analyzed using binomial logistic regression analysis with the forced entry method. Odds ratios (ORs) and 95% confidence intervals (CIs) were calculated to assess the strength of associations. Sport-stratified prevalence was calculated using the chi-squared test with 95% CIs. A 95% CI, not including 1.00, was considered statistically significant.

Site- and sex-stratified comparisons of “injuries” and “complaints” were performed using the injury proportion ratio (IPR). This ratio facilitates comparisons of relative proportions between categorical variables, which is particularly useful in injury epidemiology when exposure data are unavailable [13,10,11]. For example, an IPR comparing the proportion of knee injuries classified as “injury” with that of knee injuries classified as “complaints” would be calculated as follows:

$IPR\; knee\; &ldquo;injury&rdquo;/&ldquo;complaint&rdquo;=\frac{\left( \frac{No.of knee injuries during &ldquo;injury&rdquo;}{Total No.of injuries during &ldquo;injury&rdquo;} \right)}{\left( \frac{No.of knee injuries during &ldquo;complaint&rdquo;}{Total No.of injuries during &ldquo;complaint&rdquo;} \right)}$

Statistical analyses were conducted using SPSS (IBM SPSS Statistics for Windows, IBM Corp., Version 28.0, Armonk, NY).

## Results

The data comprised a cumulative total of 10,854 athletes (female: 4,966 {45.8%, 95% CI; 0.45-0.47}; male: 5,888 {54.2%; 95% CI, 0.53-0.55}; age, 22.0 {20.0-25.0} years), from 56 sports and 30 games (2008-2019), among whom 6,416 individuals visited our clinic (Table 1). The registered international multi-sport events are listed in Supplementary Material 1. The top 50 sports and the number of participants examined are listed in Supplementary Material 2.

**Table 1:** Demographic data of participants

International multi-sport event	Number of games	Female	Male	Total	Age
		n (%)	n (%)	n	Median (interquartile range)
Summer	17	4,043 (46.1)	4,726 (53.9)	8,769	
Olympic Games	3	851 (47.9)	924 (52.1)	1,775	24.0 [22.0-28.0]
Asia Games	3	1,430 (45.3)	1727 (54.7)	3,157	24.0 [21.0-27.0]
East Asia Games	2	409 (44.0)	520 (56.0)	929	23.0 [21.0-27.0]
Universiade	6	1,194 (45.9)	1,407 (54.1)	2,601	21.0 [20.0-21.0]
Youth Olympic Games	3	159 (51.8)	148 (48.2)	307	17.0 [16.0-17.0]
Winter	13	923 (44.3)	1,162 (55.7)	2,085	
Olympic Games	3	315 (45.5)	377 (54.5)	692	24.0 [21.0-28.0]
Asia Games	2	185 (43.5)	240 (56.5)	425	23.0 [20.0-27.0]
Universiade	6	349 (42.9)	464 (57.1)	813	20.0 [19.0-22.0]
Youth Olympic Games	2	74 (47.7)	81 (52.3)	155	15.0 [14.0-16.0]
Total	30	4966 (45.8)	5888 (54.2)	10,854	22.0 [20.0-25.0]

Registration and distribution of injuries

Prevalence of “Injuries”

A total of 2,333 “injuries” were recorded, corresponding to 21.5 “injuries” per 100 athletes. The prevalence of “injuries,” that is, the number of individual athletes with an “injury” divided by the total number of athletes examined, was 16.2% (95% CI, 0.16-0.17). Univariate analysis showed no difference in the mean age of “injured” athletes (P = 0.76). Female athletes were more prone to “injury” than male athletes (χ^2^ = 11.23, P < 0.001). In generational comparisons, “injuries” were more common in the adult group than in the youth group (χ^2^ = 5.57, P = 0.018). In binary logistic regression analyses, the female (OR, 1.21; 95% CI, 1.09-1.34) and adult group (OR, 1.35; 95% CI, 1.08-1.69) groups had a higher “injury” prevalence.

Prevalence of “Complaints”

A total of 100,027 “complaints” were registered, corresponding to 92.4 “complaints” per 100 athletes. The “complaint” prevalence was 55.3% (0.54-0.56), with only one “complaint” counted for each athlete with multiple “complaints.” In the univariate analysis, athletes with “complaints” were more likely older (P < 0.001) and female (χ^2^ = 78.70, P < 0.001). In generational comparisons, “complaints” were more common in the adult group than in the youth group (χ^2^ = 19.82, P < 0.001). The binomial logistic regression analysis showed that “complaint” prevalence was significantly higher in females (OR, 1.44; 95% CI, 1.33-1.55) and the adult group (OR, 1.50; 95% CI, 1.29-1.75).

Therefore, the prevalence of “injuries” and “complaints” was significantly higher in the female and adult groups.

Comparison of “injury” and “complaint” prevalence by sport

Prevalence of “Injuries”

The “injury” prevalence data for competitions with >100 participants are shown in Table 2.

**Table 2:** "Injury" proportion per athlete and prevalence for competitions with >100 participants The analysis was conducted using a chi-squared test. *P < 0.05, †P < 0.001. (a) “Injury” proportion per athlete: number of “injuries” (n) / registered athletes (n); how many number of “injuries” each athlete has. (b) “Injury” prevalence (%): “injured” athletes (n) / registered athletes (n) × 100. (c) Binary logistic regression analyses. (d) Baseball refers to male players only. (e) Fisher's exact test.

Sports	Registered athletes (n)			All "injuries" (n)						"Injury" proportion per athlete^ a^		
				Athletes with "injuries" (n)			Sex differences			"Injury" prevalence^ b^ (%)		
	Female	Male	Total	Female	Male	Total	OR	95% CI	χ*^2^*-value	Female	Male	Total
Grand total	4,966	5,888	10,854	1,180	1,153	2,333				0.24	0.20	0.21
				867	888	1,755	1.21 ^c^	1.09-1.34^†^^c^		17.5	15.1	16.2
Athletics	433	668	1,101	96	117	213				0.22	0.18	0.19
				70	94	164	1.18	0.84-1.65	0.91	16.2	14.1	14.9
Soccer	327	478	805	50	111	161				0.15	0.23	0.20
				43	89	132	0.66	0.45-0.98*	4.24	13.1	18.6	16.4
Volleyball	287	306	593	202	201	403				0.70	0.66	0.68
				117	122	239	1.04	0.75-1.44	0.05	40.8	39.9	40.3
Skiing	214	370	584	26	35	61				0.12	0.09	0.10
				20	28	48	1.26	0.69-2.30	0.57	9.3	7.6	8.2
Skating	243	292	535	57	49	106				0.23	0.17	0.20
(Speed/Short track)				39	40	79	1.20	0.75-1.94	0.58	16.0	13.7	14.8
Swimming	237	278	515	44	30	74				0.19	0.11	0.14
				31	24	55	1.59	0.91-2.80	2.65	13.1	8.6	10.7
Basketball	277	213	490	80	51	131				0.29	0.24	0.27
				61	42	103	1.15	0.74-1.79	0.39	22.0	19.7	21.0
Ice hockey	215	227	442	20	37	57				0.09	0.16	0.13
				18	32	50	0.56	0.30-1.03	3.61	8.4	14.1	11.3
Rugby	155	187	342	31	44	75				0.20	0.24	0.22
				24	39	63	0.70	0.40-1.22	1.63	15.5	20.9	18.4
Hockey	194	130	324	59	11	70				0.30	0.08	0.22
				44	10	54	3.52	1.70-7.29^†^	12.59	22.7	7.7	16.7
Gymnastics	208	116	324	77	41	118				0.37	0.35	0.36
				57	29	86	1.13	0.67-1.90	0.22	27.4	25.0	26.5
Judo	160	156	316	37	37	74				0.23	0.24	0.23
				29	26	55	1.11	0.62-1.98	0.12	18.1	16.7	17.4
Badminton	139	135	274	32	34	66				0.23	0.25	0.24
				30	26	56	1.15	0.64-2.08	0.23	21.6	19.3	20.4
Fencing	136	138	274	28	12	40				0.21	0.09	0.15
				22	10	32	2.47	1.12-5.44*	5.3	16.2	7.2	11.7
Water polo	104	161	265	17	25	42				0.16	0.16	0.16
				11	19	30	0.88	0.40-1.94	0.09	10.6	11.8	11.3
Baseball ^d^	-	242	242	-	23	23				-	0.10	0.10
				-	21	21		-		-	8.7	8.7
Cycling	79	143	222	11	18	29				0.14	0.13	0.13
				9	18	27	0.89	0.38-2.09	0.07	11.4	12.6	12.2
Handball	120	100	220	41	32	73				0.34	0.32	0.33
				28	21	49	1.15	0.60-2.17	0.17	23.3	21.0	22.3
Figure skating	93	100	193	28	27	55				0.30	0.27	0.28
				19	20	39	1.03	0.51-2.07	0.01	20.4	20.0	20.2
Table tennis	88	77	165	16	11	27				0.18	0.14	0.16
				14	11	25	1.14	0.48-2.67	0.08	15.9	14.3	15.2
Weightlifting	78	73	151	31	28	59				0.40	0.38	0.39
				23	21	44	1.04	0.51-2.09	0.01	29.5	28.8	29.1
Tennis	67	63	130	12	5	17				0.18	0.08	0.13
				12	4	16	3.22	0.98-10.58	0.06	17.9	6.3	12.3
Rifle shooting	63	66	129	10	9	19				0.16	0.14	0.15
				9	8	17	1.21	0.44-3.36	0.13	14.3	12.1	13.2
Wrestling	42	83	125	29	31	60				0.69	0.37	0.48
				18	27	45	1.56	0.72-3.34	1.29	42.9	32.5	36.0
Snowboarding	49	61	110	4	7	11				0.08	0.11	0.10
				4	7	11	0.69^e^	0.19-2.49^e^	-	8.2	11.5	10.0
Rowing	35	69	104	4	9	13				0.11	0.13	0.13
				4	8	12	0.98^e^	0.28-3.52^e^	-	11.4	11.6	11.5
Golf	50	53	103	5	3	8				0.10	0.06	0.08
				4	3	7	1.45^e^	0.31-6.83^e^	-	8.0	5.7	6.8

The “injury” prevalence (number of injured athletes / number of registered athletes) was significantly higher in males than in females for soccer but was higher in females for hockey and fencing. No statistically significant sex difference was observed in the other sports (volleyball: 40.3% {95% CI, 0.36-0.44}; wrestling: 36.0% {0.28-0.44}; weightlifting: 29.1% {0.22-0.36}; gymnastics: 26.5% {0.22-0.31}; and handball: 22.3% {0.17-0.28}).

The number of “injuries” per individual was expressed as the proportion of “injuries” per athlete (number of injuries / number of registered athletes). In other words, this indicates how many injuries one athlete has. Sports with higher prevalence also demonstrated a greater number of 'injuries' per athlete. Although the “injury” proportion per athlete was higher in females for most sports, this proportion was higher in males for soccer, ice hockey, rugby, and rowing.

Prevalence of “Complaints”

The “complaint” prevalence data for competitions with >100 participants are shown in Table 3.

**Table 3:** "Complaint" proportion per athlete and prevalence for competitions with >100 participants The analysis was conducted using a chi-squared test. *P < 0.05, †P < 0.001. (a) “Complaint” proportion per athlete: “complaints” (n) / registered athletes (n); how many number of “complaints” each athlete has. (b) “Complaint” prevalence (%): Athletes with “complaints” (n) / registered athletes (n) × 100. (c) Binary logistic regression analyses. (d) Baseball refers to male players only.

Sports	Registered athletes (n)			All "complaints" (n)						"Complaint" proportion per athlete^ a^		
				Athletes with "complaints" (n)			Sex differences			"Complaint" prevalence ^b^ (%)		
	Female	Male	Total	Female	Male	Total	OR	95% CI	χ^2^-value	Female	Male	Total
Grand total	4,966	5,888	10,854	5,173	4,854	10,027				1.04	0.82	0.92
				2,978	3,029	6,007	1.44 ^c^	1.33-1.55^†^^c^		60.0	51.4	55.3
Athletics	433	668	1101	407	535	942				0.94	0.80	0.86
				258	347	605	1.36	1.07-1.74*	6.19	59.6	51.9	55.0
Soccer	327	478	805	240	312	552				0.73	0.65	0.69
				172	238	410	1.12	0.84-1.48	0.61	52.6	49.8	50.9
Volleyball	287	306	593	538	553	1091				1.87	1.81	1.84
				234	237	471	1.29	0.86-1.92	1.51	81.5	77.5	79.4
Skiing	214	370	584	163	177	340				0.76	0.48	0.58
				104	134	238	1.67	1.18-2.34*	8.61	48.6	36.2	40.8
Skating	243	292	535	224	214	438				0.92	0.73	0.82
(Speed/Short track)				117	144	261	0.95	0.68-1.34	0.07	48.1	49.3	48.8
Swimming	237	278	515	212	181	393				0.89	0.65	0.76
				127	122	249	1.48	1.04-2.09*	4.82	53.6	43.9	48.3
Basketball	277	213	490	358	214	572				1.29	1.00	1.17
				193	138	331	1.25	0.85-1.83	1.31	69.7	64.8	67.6
Ice hockey	215	227	442	138	141	279				0.64	0.62	0.63
				95	98	193	1.04	0.72-1.52	0.05	44.2	43.2	43.7
Rugby	155	187	342	216	224	440				1.39	1.20	1.29
				120	130	250	1.50	0.92-2.45	2.69	77.4	69.5	73.1
Hockey	194	130	324	238	99	337				1.23	0.76	1.04
				127	63	190	2.02	1.28-3.17*	9.28	65.5	48.5	58.6
Gymnastics	208	116	324	293	171	464				1.41	1.47	1.43
				160	84	244	1.27	0.76-2.14	0.81	76.9	72.4	75.3
Judo	160	156	316	220	160	380				1.38	1.03	1.20
				114	93	207	1.68	1.05-2.68*	4.73	71.3	59.6	65.5
Badminton	139	135	274	129	94	223				0.93	0.70	0.81
				82	62	144	1.69	1.05-2.73*	1.69	59.0	45.9	52.6
Fencing	136	138	274	151	94	245				1.11	0.68	0.89
				86	61	147	2.17	1.34-3.52*	9.98	63.2	44.2	53.6
Water polo	104	161	265	120	124	244				1.15	0.77	0.92
				68	85	153	1.69	1.02-2.81*	4.1	65.4	52.8	57.7
Baseball ^d^	-	242	242	-	135	135				-	0.56	0.56
				-	105	105	-	-	-	-	43.4	43.4
Cycling	79	143	222	61	86	147				0.77	0.60	0.66
				45	68	113	1.46	0.84-2.54	1.80	57.0	47.6	50.9
Handball	120	100	220	171	134	305				1.43	1.34	1.39
				89	65	154	1.55	0.87-2.76	2.18	74.2	65.0	70.0
Figure skating	93	100	193	99	88	187				1.06	0.88	0.97
				58	55	113	1.36	0.76-2.41	1.08	62.4	55.0	58.5
Table tennis	88	77	165	64	60	124				0.73	0.78	0.75
				44	37	81	1.08	0.59-1.99	0.06	50.0	48.1	49.1
Weightlifting	78	73	151	143	106	249				1.83	1.45	1.65
				69	54	123	2.70	1.13-6.44*	5.24	88.5	74.0	81.5
Tennis	67	63	130	56	51	107				0.84	0.81	0.82
				33	35	68	0.78	0.39-1.55	0.52	49.3	55.6	52.3
Rifle shooting	63	66	129	48	35	83				0.76	0.53	0.64
				33	28	61	1.49	0.75-2.99	1.28	52.4	42.4	47.3
Wrestling	42	83	125	74	123	197				1.76	1.48	1.58
				32	58	90	1.38	0.59-3.23	0.55	76.2	69.9	72.0
Snowboarding	49	61	110	40	48	88				0.82	0.79	0.80
				26	34	60	0.90	0.42-1.91	0.08	53.1	55.7	54.5
Rowing	35	69	104	23	40	63				0.66	0.58	0.61
				14	29	43	0.92	0.40-2.11	0.04	40.0	42.0	41.3
Golf	50	53	103	42	20	62				0.84	0.38	0.60
				27	15	42	2.97	1.32-6.73*	7.04	54.0	28.3	40.8

The “complaint” prevalence (number of athletes with “complaints” /number of registered athletes) was higher in females for athletics, skiing, swimming, hockey, judo, badminton, fencing, water polo, weightlifting, and golf. There was no significant sex difference in the other sports. The highest levels of “complaints” (over 70%) were 81.5% (0.75-0.88) in weightlifting, 79.4% (0.76-0.83) in volleyball, 75.3% (0.710.80) in gymnastics, 73.1% (0.68-0.78) in rugby, 73.1% (0.68-0.78) in hockey, and 72.0% (0.64-0.80) in wrestling.

The “complaint” proportion per athlete (number of “complaints” / number of registered athletes), or the number of “complaints” per individual, was high in volleyball (1.84), weightlifting (1.65), wrestling (1.58), gymnastics (1.43), and handball (1.39), among others, compared to the mean proportion of 0.92. Indeed, >70% of athletes in weightlifting, volleyball, gymnastics, rugby, wrestling, and handball had multiple “complaints.”

Location of injuries, stratified by sex

**Figure 1:**
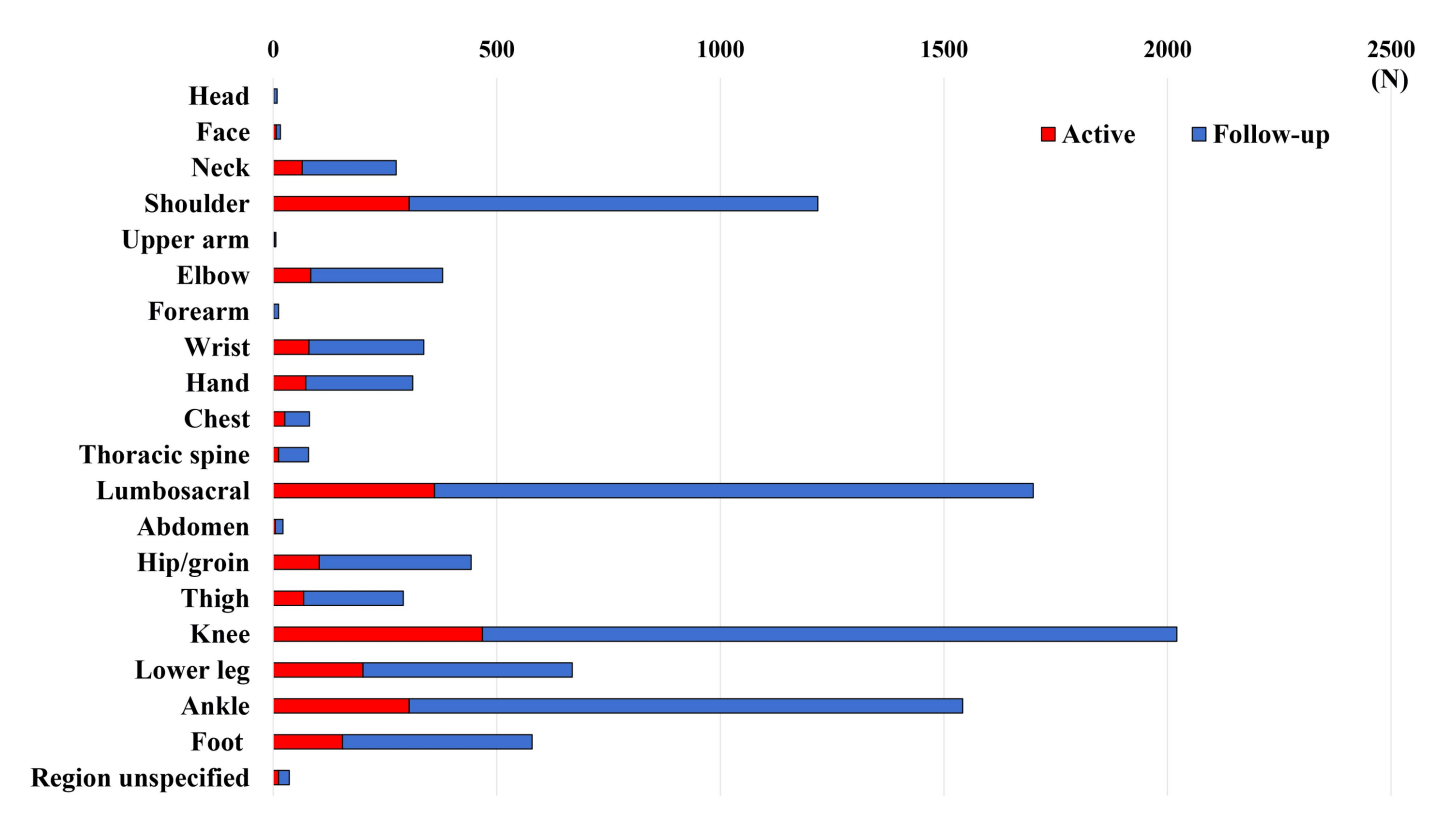
Injury locations “Active” or “follow-up” injuries were categorized based on severity. Injuries classified as “active” (red) were defined as “injuries” in this study. The sum of both “active” (red) and “follow-up” (blue) injuries is considered a "complaint.”

The aggregates for injuries at each location are shown in Figure 1.

Of the 2,333 “injuries,” the most commonly injured anatomical location was the knee (468 “injuries,” 20.1% {95% CI 0.18-0.22}), followed by the lumbosacral (361, 15.5% {0.14-0.17}), ankle (304, 13.0% {0.12-0.14}), and shoulder (304, 13.0% {0.12-0.14}) (Table 4).

**Table 4:** Representative locations and diagnoses of “injuries” The analysis was conducted using a chi-squared test. *P < 0.05, †P < 0.001. (a) IPR, injury proportion ratio. (b) F/M, female/male.

Location	Female	Male	Total	Sex differences			
	n	n	n (%)	IPR ^a^F/M ^b^	95% CI	χ^2^-value	P-value
Shoulder	142	162	304 (13.0)	0.84	0.66-1.07	2.09	0.15
Shoulder tendinopathy	31	45	76 (3.3)	0.66	0.42-1.06	3.01	0.08
Lumbosacral	198	163	361 (15.5)	1.23	0.98-1.53	3.11	0.08
Lower back pain	64	56	120 (5.1)	1.12	0.78-1.62	0.38	0.54
Knee	206	262	468 (20.1)	0.72	0.59-0.88	10.08	0.001*
Patellar tendinopathy	34	75	105 (4.5)	0.43	0.28-0.65	17.19	<0.001^†^
Ankle	146	158	304 (13.0)	0.89	0.70-1.13	0.91	0.34
Ankle ligament tear/sprain	50	56	106 (4.5)	0.87	0.59-1.28	0.52	0.47
Chronic ankle instability	37	23	60 (2.6)	1.59	0.94-2.69	3.03	0.08
Grand total	1,180	1,153	2,333	-	-	-	-

Among the 10,027 “complaints,” 2,021 (20.2% {0.19-0.21}) involved the knee, followed by the lumbosacral (1,700, 17.0% {0.16-0.18}), ankle (542, 15.4% {0.15-0.16}), and shoulder (1,218, 12.1% {0.11-0.13}) (Table 5).

**Table 5:** Representative locations and diagnoses of “complaints” The analysis was conducted using a chi-squared test. *P < 0.05, †P < 0.001. (a) IPR, injury proportion ratio. (b) F/M, female/male.

Location	Female	Male	Total	Sex differences			
	n	n	n (%)	IPR ^a^F/M ^b^	95% CI	χ^2^-value	P-value
Shoulder	600	618	1,218 (12.1)	0.90	0.80-1.01	3.01	0.08
Shoulder tendinopathy	137	141	278 (2.8)	0.91	0.72-1.15	0.61	0.43
Lumbosacral	860	840	1,700 (17.0)	0.95	0.86-1.06	0.82	0.36
Lower back pain	358	333	691 (6.9)	1.01	0.87-1.18	0.01	0.91
Knee	1,035	986	2,021 (20.2)	0.98	0.89-1.08	0.15	0.70
Patellar tendinopathy	177	230	407 (4.1)	0.71	0.58-0.87	11.15	<0.001^†^
Ankle	842	700	1,542 (15.4)	1.15	1.04-1.29	6.63	0.01*
Ankle ligament tear/sprain	236	205	441 (4.4)	1.08	0.90-1.31	0.68	0.41
Chronic ankle instability	361	229	590 (5.9)	1.52	1.28-1.80	23.11	<0.001^†^
Grand total	5,173	4,854	10,027	-	-	-	-

We compared the IPR for the four sites with the highest number of “injuries”: the knee, lumbosacral, ankle, and shoulder (IPR knee injury/complaint {I/C}: 0.99 {95% CI, 0.89-1.11}, χ^2^ = 0.01, P = 0.92); IPR lumbosacral I/C: 0.90 {0.79-1.02}, χ^2^ = 2.99, P = 0.08; IPR ankle I/C: 0.82 {0.72-0.94}, χ^2^ = 8.21, P = 0.004; and IPR shoulder I/C: 1.08 {0.95-1.24}, χ^2^ = 1.37, P = 0.24). The proportion of ankle injuries was higher among the “complaint” than the “injury” category (Table 6).

**Table 6:** IPR injury/complaint of representative locations and diagnoses The analysis was conducted using a chi-squared test. *P < 0.05, †P < 0.001. (a) IPR, injury proportion ratio. (b) I/C, injury/complaint.

Location	IPR ^a^ I/C ^b^	95% CI	χ^2^-value	P-value
Shoulder	1.08	0.95-1.24	1.37	0.24
Shoulder tendinopathy	1.19	0.92-1.53	1.67	0.20
Lumbosacral	0.90	0.79-1.02	2.99	0.08
Lower back pain	0.73	0.60-0.89	9.43	0.002*
Knee	0.99	0.89-1.11	0.01	0.92
Patellar tendinopathy	1.11	0.89-1.39	0.93	0.34
Ankle	0.82	0.72-0.94	8.21	0.004*
Ankle ligament tear/sprain	1.03	0.83-1.28	0.07	0.80
Chronic ankle instability	0.42	0.32-0.55	41.68	< .001^ †^

The most common diagnoses of ankle injuries were chronic ankle instability (CAI) and ankle ligament tear/sprain (IPR CAI I/C, 0.42 {95% CI 0.32-0.55}, χ^2^ = 41.68, P < 0.001; IPR ankle ligament tear/sprain I/C, 1.03 {0.83-1.28}, χ^2^ = 0.07, P = 0.80). Thus, CAI was more commonly registered as a “complaint” than “injury.”

When stratified by injury location and sex, males presented with significantly more knee “injury” but not shoulder, lumbosacral, or ankle “injury” (Table 4). “Complaints” pertaining to the ankle, but not the shoulder, lumbosacral, or knee, were significantly more common in females than in males (Table 5). Patellar tendinopathy, common in the knee joint, was a more common “injury” and “complaint” in males, whereas CAI, common in the ankle joint, was a significantly more common “complaint” in females.

## Discussion

The JISS medical check-up

This is the first study to report out-of-competition injuries in elite Japanese athletes across sports. Outside the games, no previous study has reported on injuries in elite Asian athletes across sports. Before competitions, our clinic conducts medical check-ups of JOC-designated athletes and provides feedback to the athletes and medical staff of the associated sports organization. The medical examinations were conducted at the same medical institution by well-trained orthopedic surgeons, certified as domestic sports specialists, based on common standards.

Since the data were cross-sectional and derived from medical check-ups, they do not accurately reflect the incidence of injuries over a defined period. Additionally, excluding previously healed, uncomplicated injuries may have introduced a bias, leading to the overrepresentation of overuse or chronic injuries relative to acute trauma. Our evaluation encompassed injuries that could result in performance loss, from severe injuries that restrict sports participation to minor or chronic injuries that do not necessitate medical intervention or training restrictions, even if not classified as 'time-loss' injuries [12]. To provide insights for medical staff on the characteristics of injuries and to enhance support for high-performance athletes in international competitions, we assessed both acute and chronic injuries, as well as complaints, in elite Japanese athletes.

Prevalence of injuries

Our study found that 21.5 “injuries” per 100 athletes, corresponding to a prevalence of 16.2%. In contrast, 92.4 “complaints” per 100 athletes, corresponding to a prevalence of 55.3%.

During the London Olympic Summer Games, the overall injury rate was 12.9 injuries per 100 registered athletes, and at least one injury occurred in 11% of the athletes [4]. In the Rio Olympic Summer Games, 9.8 injuries occurred per 100 participating athletes, and 8% of the athletes sustained at least one injury [7]. During the PyeongChang Olympic Winter Games, 12.6 injuries occurred per 100 exposed athletes over the 17-day period, and 12% of the athletes reported at least one injury [6]. According to Asian data, during the 2018 Summer Asian Games, at least one injury occurred in 15.5% of athletes on the Korean team [14]. While these previous studies were conducted over a limited competitive period of up to three weeks, our current study covered non-competitive periods and examined prevalence, not incidence, so the “injury” prevalence may have been slightly higher. The results of this cross-sectional study are more similar to those for the Korean team during the Asian Summer Games than the overall data for the Olympic Games, likely influenced by differences in the level of competition and ethnicity.

In contrast, Clarsen et al. [15] reported that among athletes preparing for the Olympic and Paralympic Games in Norway, 36% had health problems at any given time. This is the only study on the incidence of health problems among athletes preparing for the Olympic and Paralympic Games, and it used a prospective questionnaire survey. The high “complaint” prevalence observed in the present study may be attributable to differences in the study population and specialist identification of symptoms that were not self-reported by the athletes in the questionnaire.

Sex, sport, and location

The prevalence of both “injuries” and “complaints” was significantly higher in females (OR, 1.19 for “injuries” and 1.44 for “complaints”), with “complaints” showing a slightly stronger association with females.

The incidence of injuries during the Vancouver 2010 Olympic Winter Games was higher in females than in males (rate ratio, 1.4; 95% CI, 1.1-1.8) [5]. In contrast, at the 2016 Summer Olympics Games, the overall incidence of injuries was almost equal for females and males (risk ratio, 0.99; 95% CI, 0.87-1.11); however, females had a significantly higher incidence of injuries in some events [7]. Although the present study combined data from the winter and summer competitions, the results were similar. An examination of sex differences in orthopedic research showed that only 34% (241 of 712) of studies published in 2016 included sex as a variable in multifactorial statistical models; of these, 39% reported differences in outcomes between females and males [16]. Sex differences in sports injuries are crucial considerations for developing preventive measures; however, most sports medicine studies have predominantly focused on male participants [16], highlighting the necessity for more research that includes female athletes.

Injury prevalence across sports has been rarely reported. A Danish study of general athletes reported that the prevalence of injuries was higher during running, soccer, and strength training among adults [17]. In the Pyeongchang Winter Olympics, the highest incidence of injuries was reported in the ski halfpipe (28%), snowboard cross (26%), ski cross (25%), snowboard slopestyle (21%), and aerials (20%) [6]. At the London Summer Olympics, the highest risk of injury was observed in taekwondo, soccer, bicycle motocross (BMX) cycling, handball, mountain biking, track and field, and athletics [4]. In the Rio Summer Olympics, the highest incidence of injury was reported in BMX cycling (37.5 injuries per 100 athletes {95% CI 20.2-54.8}), boxing (30.1 {23.7-36.4}), mountain bike cycling (23.8 {13.1-34.4}), and taekwondo (23.6 {15.2-32.1}) [7]. During the Asian Summer Games, the Korean team reported the most injuries in climbing and sepak takraw [14]. Among college students in 15 sports, soccer had the highest injury rates in both practice and competition [18].

In this study, injuries were more prevalent in volleyball, weightlifting, and wrestling. Our clinic provides outpatient care in addition to medical check-ups. During the check-ups, we also review outpatient records. However, referencing these records carries the risk that athletes with frequent outpatient visits may have more injuries documented, potentially skewing the data. On the other hand, the results of our medical examinations may offer a more accurate evaluation than diagnoses made at other medical institutions. Furthermore, although racial differences should be considered in this field, they cannot be accounted for due to the lack of comparable studies on this topic.

The knee, lumbosacral, ankle, and shoulder were the most common sites of “injuries” and “complaints” in that order. During the Pyeongchang Winter Olympics, the most common injuries occurred in the knee, followed by the ankle joint, hands/fingers, and lower back [6]. During the Rio Summer Olympics, the most frequently injured sites were the knees (n = 130), thighs (n = 108), ankles (n = 103), face (n = 94), and lower limbs (n = 90) [7]. In a survey conducted in Norway prior to the 2012 Summer Olympic and Paralympic Games, the combined incidence of acute and overuse injuries occurred in the following order: knee > lower back > shoulder > foot > ankle [11]. Although the present study differs from previous studies in that it considered the preparation phases for both the summer and winter games, the knee was still the most common site of injury.

Comparing the relative importance of “injury” and “complaint” by site, we found no significant differences for the knee, hip, and shoulder, but the ankle had a higher proportion of “complaint” than “injury” (IPR ankle I/C, 0.82 {95% CI, 0.72-0.94, P = 0.004}). Acute ankle sprains are most frequently reported in college athletes in the United States [19]. Up to 70% of those who suffer a lateral ankle sprain may develop CAI within a short period after the initial injury. In this study, CAI was a frequently diagnosed condition and was more common among “complaint” than “injury,” reflecting the potential for ankle sprains to progress or persist as a “complaint” of CAI. Additionally, in gender differences, ankle “complaint” was significantly more common in females than in males. Similarly, previous studies showed that the incidence of acute ankle sprain, the most common ankle joint injury, was higher in females than in males (13.6 vs. 6.9/1000) [20], and CAI was approximately twice as common in females (32%) than in males (17%) athletes [21]. These findings suggest the importance of considering sex differences in injury prevention strategies [22] and rehabilitation protocols for ankle injuries [23]. In our study, knee “injury” was significantly more common in males than in females. Using the National High School Sports-Related Injury Surveillance System, Swenson et al. [24] demonstrated that the incidence of knee injury was significantly higher in females than in males in high school students in comparable sports. For example, anterior cruciate ligament (ACL) injury is the most common knee injury in females, but it varies by sport. In soccer and basketball, ACL injuries are three times more prevalent in females than in males [25]. However, in a long-term study of the general population, the incidence of ACL injury was higher in males than in females (81.7 vs. 55.3/100,000, P < 0.001) and that the incidence peaked at 19-25 years of age for males (241.0/100,000) and 14-18 years for females (227.6/100,000) [26]. Thus, even for ACL injuries alone, sex differences in injury incidence and predilection vary by age and competition level. Swenson et al. reported a higher incidence of knee injuries in females [24], reflecting the high number of ACL injuries in high-school-aged females, which differed from the age range covered in the present study. In contrast, males had more combined injuries of the meniscus [27], and patellar tendinopathy was more common in male volleyball and basketball players (OR, 2.0; 95% CI, 1.1-3.5) [28]. In this study, patellar tendinopathy was also more common in males and the most commonly registered knee injury, followed by lateral meniscal injury. We assumed that knee “injury” was more common in males because of the higher rates of patellar tendinopathy and meniscal injury.

Although the overall prevalence of injury was higher among female athletes, the results varied depending on anatomical site and type of sport. While preventive measures targeting female athletes in specific sports have been reported [29], knowledge regarding strategies to reduce overall injury rates - rather than focusing on individual sports or specific injuries - remains limited. Identifying the focus for preventive strategies remains controversial. However, the injury characteristics in this study can inform the development of more effective injury prevention strategies and high-performance support plans for elite Japanese athletes participating in international competitions.

Limitations

First, diagnosis primarily relied on clinical records due to the time demands of physically evaluating numerous patients. Although the attending physician could request imaging studies such as US, plain X-rays, CT, or MRI when necessary, the decision was left to the physician, which may limit diagnostic accuracy due to inconsistent criteria. Second, “total attendance" refers to the aggregate number of individuals participating in medical check-ups; each visit was counted as a separate athlete if they participated multiple times. Therefore, this study does not account for potential repeat visits. There is room for discussion on how to interpret cases where the same athlete continues to present with the same chronic condition in the aggregation of multiple cross-sectional surveys. Addressing this issue will be a task for future research. Third, the study population comprised only elite Japanese athletes who were selected as candidates for international multi-sports games rather than all athletes participating in all sports. Finally, the classification of "active" and "follow-up" injuries was subjective and would vary among physicians and sports.

## Conclusions

This is the first cross-sectional analysis of injuries in Asian international athletes outside competitive periods. Among Japanese international athletes, 21.5 “injuries” per 100 athletes, “injuries” that required treatment, and 92.4 “complaints” per 100 athletes that required treatment or observation. More than half of patients had “complaints” of at least one musculoskeletal symptom. “Injury” prevalence was higher in females and adults than in males and youths, respectively. Ankle “complaint” was significantly more common than ankle “injury.” Knee “injury” was significantly more common in males, whereas ankle “complaint” was significantly more common in females, indicating sex-specific injury patterns in terms of site and severity. In future studies, we aim to further examine injury patterns across different generations, competition levels, and sports types while considering how these trends may evolve over time. This will aid in developing targeted prevention and treatment strategies, ultimately improving athlete safety and performance.

## Appendices

**Table 7:** List of registered games

Summer	Winter
Olympic Games	Olympic Games
Beijing 2008	Vancouver 2010
London 2012	Sochi 2014
Rio de Janeiro 2016	PyeongChang 2018
Asia Games	Asia Games
Guangzhou 2010	Astana 2011
Incheon 2014	Sapporo 2017
Jakarta 2018	Universiade
East Asia Games	Harbin 2009
Hong kong 2009	Erzurum 2011
Tianjin 2013	Tolentino 2013
Universiade	Granada 2015
Belgrade 2009	Almaty 2017
Shenzhen 2011	Krasnoyarsk 2019
Kazan 2013	Youth Olympic Games
Gwangju 2015	Innsbruck 2012
Taipei 2017	Lillehammer 2016
Naples 2019	
Youth Olympic Games	
Singapore 2010	
Nanjing 2014	
Buenos Aires 2018	

**Table 8:** List of top 50 sports and number of participants

Sports	Female	Male	Total
	n (%)	n (%)	n
Grand total	4,966 (45.8)	5,888 (54.2)	10,854
Athletics	433 (39.3)	668 (60.7)	1,101
Soccer	327 (40.6)	478 (59.4)	805
Volleyball	287 (48.4)	306 (51.6)	593
Skiing	214 (36.6)	370 (63.4)	584
Skating (speed/short track)	243 (45.4)	292 (54.6)	535
Swimming	237 (46.0)	278 (54.0)	515
Basketball	277 (56.5)	213 (43.5)	490
Ice hockey	215 (48.6)	227 (51.4)	442
Rugby	155 (45.3)	187 (54.7)	342
Hockey	194 (59.9)	130 (40.1)	324
Gymnastics	208 (64.2)	116 (35.8)	324
Judo	160 (50.6)	156 (49.4)	316
Badminton	139 (50.7)	135 (49.3)	274
Fencing	136 (49.6)	138 (50.4)	274
Water polo	104 (39.2)	161 (60.8)	265
Baseball	0 (0.0)	242 (100.0)	242
Cycling	79 (35.6)	143 (64.4)	222
Handball	120 (54.5)	100 (45.5)	220
Figure skating	93 (48.2)	100 (51.8)	193
Table tennis	88 (53.3)	77 (46.7)	165
Weightlifting	78 (51.7)	73 (48.3)	151
Tennis	67 (51.5)	63 (48.3)	130
Rifle shooting	63 (48.8)	66 (51.2)	129
Wrestling	42 (33.6)	83 (66.4)	125
Snowboarding	49 (44.5)	61 (55.5)	110
Rowing	35 (33.7)	69 (66.3)	104
Golf	50 (48.5)	53 (51.5)	103
Saling	37 (37.4)	62 (62.6)	99
Equestrian	22 (22.4)	76 (77.6)	98
Archery	43 (50.6)	42 (49.4)	85
Curling	50 (59.5)	34 (40.5)	84
Canoeing	32 (38.1)	52 (61.9)	84
Softball	83 (100.0)	0 (0.0)	83
Beach volleyball	42 (51.9)	39 (48.1)	81
Taekwondo	37 (47.4)	41 (52.6)	78
Artisitic swimming	73 (100.0)	0 (0.0)	73
Diving	35 (49.3)	36 (50.7)	71
Biathlon	30 (49.2)	31 (50.8)	61
Kabaddi	23 (38.3)	37 (61.7)	60
Dragon boat	11 (18.3)	49 (81.7)	60
Bowling	30 (50.0)	30 (50.0)	60
Sepak takraw	27 (46.6)	31(53.4)	58
Triathlon	31 (56.4)	24 (43.6)	55
Dance sports	23 (50.0)	23 (50.0)	46
Trampoline	14 (30.4)	32 (69.6)	46
Boxing	7 (15.2)	39 (84.8)	46
Modern pentathlon	21 (45.7)	25 (54.3)	46
Martial arts tai chi	21 (46.7)	24 (53.3)	45
Karate	21 (50.0)	21 (50.0)	42
